# Measuring neuronal activity with diffuse correlation spectroscopy: a theoretical investigation

**DOI:** 10.1117/1.NPh.8.3.035004

**Published:** 2021-08-05

**Authors:** Xiaojun Cheng, Edbert J. Sie, Stephanie Naufel, David A. Boas, Francesco Marsili

**Affiliations:** a Boston University, Neurophotonics Center, Department of Biomedical Engineering, Massachusetts, United States; b Facebook Reality Labs Research, Menlo Park, California, United States

**Keywords:** diffuse correlation spectroscopy, Monte Carlo, neuronal activation, neuronal cell motion, hemodynamics

## Abstract

**Significance**: Diffuse correlation spectroscopy (DCS) measures cerebral blood flow non-invasively. Variations in blood flow can be used to detect neuronal activities, but its peak has a latency of a few seconds, which is slow for real-time monitoring. Neuronal cells also deform during activation, which, in principle, can be utilized to detect neuronal activity on fast timescales (within 100 ms) using DCS.

**Aims**: We aim to characterize DCS signal variation quantified as the change of the decay time of the speckle intensity autocorrelation function during neuronal activation on both fast (within 100 ms) and slow (100 ms to seconds) timescales.

**Approach**: We extensively modeled the variations in the DCS signal that are expected to arise from neuronal activation using Monte Carlo simulations, including the impacts of neuronal cell motion, vessel wall dilation, and blood flow changes.

**Results**: We found that neuronal cell motion induces a DCS signal variation of $∼10^{-5}$. We also estimated the contrast and number of channels required to detect hemodynamic signals at different time delays.

**Conclusions**: From this extensive analysis, we do not expect to detect neuronal cell motion using DCS in the near future based on current technology trends. However, multi-channel DCS will be able to detect hemodynamic response with sub-second latency, which is interesting for brain–computer interfaces.

## 1 Introduction

Diffuse correlation spectroscopy (DCS) is an optical imaging method that measures blood flow non-invasively and continuously. It quantifies a blood flow index by measuring the temporal autocorrelation function of the speckle intensity fluctuations of diffusive light remitted from tissue.^1^^–^^4^ A change in tissue dynamics results in a change of the decay time of the temporal autocorrelation function. Thus, DCS can be utilized to detect tissue dynamics arising from neuronal activities. The variation of the decay time is often only attributed to a change in cerebral blood flow (CBF).^5^^,^^6^ The peak of the CBF often occurs at a time delay of a few seconds with respect to the onset of neuronal activation, which is slow and not feasible for real-time monitoring of brain activation in applications such as brain–computer interfaces.

**Fig. 1:**
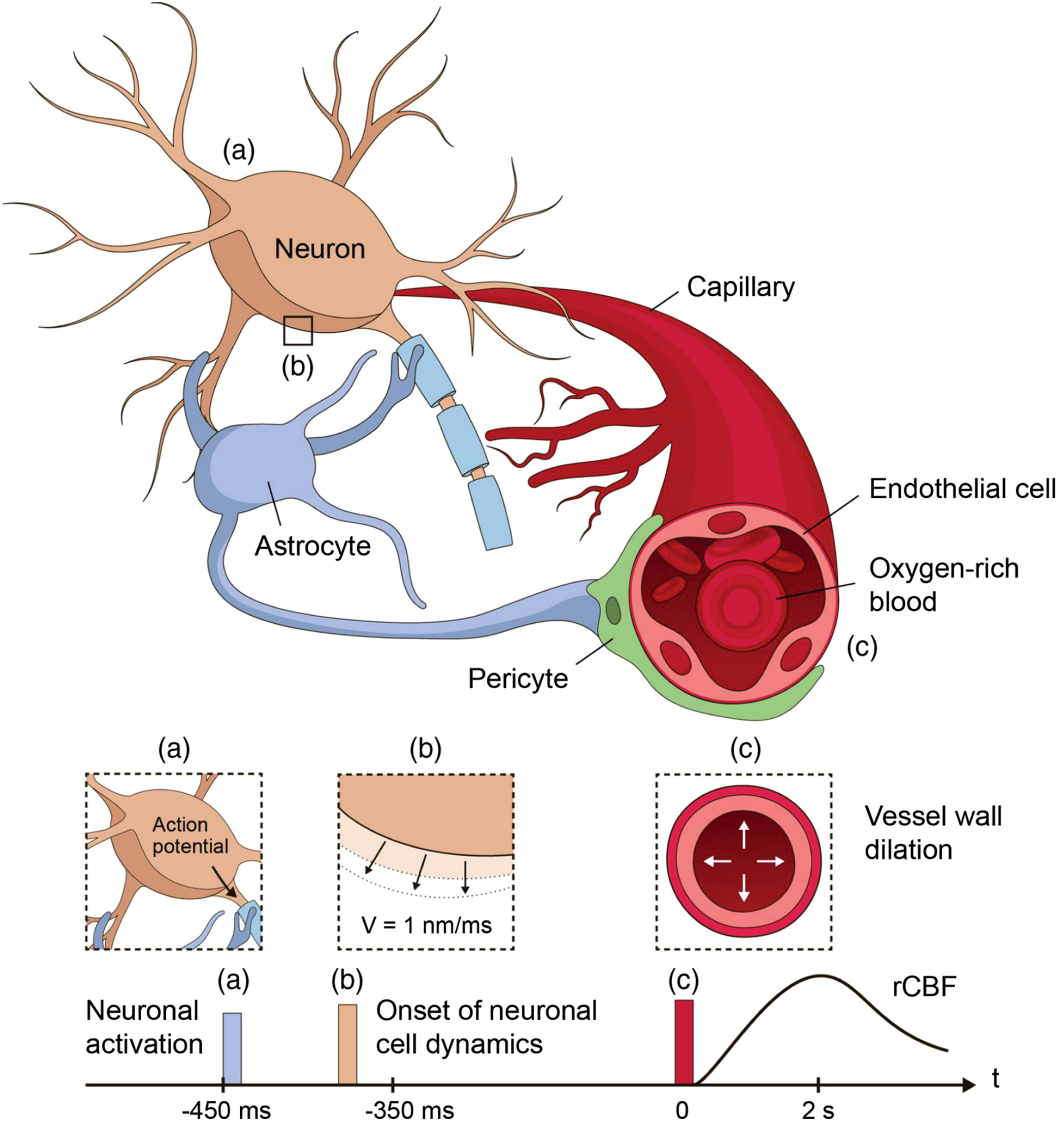
Illustration of neurovascular coupling and the onsets of neuronal activation, neuronal cell dynamics, and vessel wall dynamics. Upon brain activation, the neuron generates an action potential (a) that leads to a deformation of the neuron cell body that potentially relates to the exchange of ions through the cell membrane. (b) The onset of this neuronal cell dynamics occurs on the order of 100 ms timescale. Meanwhile, the neuron sends the signal mediated by astrocytes and pericytes to trigger blood vessel wall dilation. (c) The blood vessel wall movement leads to hemodynamic changes in the CBV and CBF. Such neurovascular coupling dynamics contribute to motions that, in principle, could be picked up by the DCS signal.

Other mechanisms that cause tissue dynamics due to neuronal activation can also contribute to a change in the DCS signal. Studies have demonstrated that the optical properties of brain tissue vary due to neuronal activation.^7^^,^^8^ The neural mechanisms that may contribute to the optical signal change may be related to the firing of action potentials. During an action potential, ions are exchanged through the membrane of the neuron. This may cause a change in the neuron shape (e.g., swelling as ions enter the cell), and one hypothesis is that these changes contribute to phase changes of light as it reflects or passes through the shifting cells.^9^^–^^12^ This fast optical signal associated with brain activation has been measured *in vivo*,^13^^–^^15^ but negative results have also been reported.^15^^,^^16^ Reported delay time of the cell dynamics with respect to the onset of neuronal activation ranges from within 1 to 100 ms,^12^^,^^15^ and our recent *in vivo* mouse brain measurements have demonstrated that most of the values of the delay time are within 100 ms,^17^ as illustrated in Fig. 1. Apart from changes to the neurons themselves, cells such as pericytes and glial cells may also reshape to support neuronal activity. In addition to direct cellular signals, neurovascular coupling causes blood vessels to dilate during neuronal activation, which delivers more oxygen to the excited region. This hemodynamic response causes a change in the phase of light scattered from the vessels, as well as cerebral blood volume (CBV) and CBF variations. The onset of hemodynamic changes has a typical delay time of 450 ms with respect to the onset of neuronal activation,^18^ which is a slower timescale compared with fast cellular signals. All of these mechanisms can result in changes in the DCS signal; as such, DCS can potentially provide important measures of real-time neuronal activity non-invasively. A comprehensive analysis of the DCS signal variation induced by neuronal activity will benefit the instrument development of highly sensitive DCS systems to study brain function.

We extensively analyzed the DCS signal variation due to neuronal activation by taking into account all of the above-mentioned mechanisms. Specifically, our model considers the impact of neuronal cell movement, blood vessel wall dilation, and blood flow and volume changes related to neuronal activities. We have found that the DCS signal change induced by neuronal cell dynamics is beyond the sensitivity of currently available DCS systems. However, sub-second detection of neuronal activation utilizing the early behavior of hemodynamics-induced DCS signal variations is technological feasible with SPAD cameras currently under development.^19^ This analysis of the mechanisms that underlie the DCS signal variation is important to the development of highly sensitive DCS systems for various applications, including studies of brain functions, monitoring of brain states at the bedside, and brain–computer interfaces.

## 2 Methods

In this section, we describe the use of Monte Carlo simulations to calculate the DCS signal variations induced by neuronal cell dynamics and hemodynamics. Other mechanisms that could contribute to DCS signal changes are also discussed. The calculation of the noise is also presented to obtain the contrast-to-noise ratio (CNR) to quantify the performance of a particular measurement.

### 2.1 Monte Carlo Simulations and the Calculation of DCS Signals

**Fig. 2:**
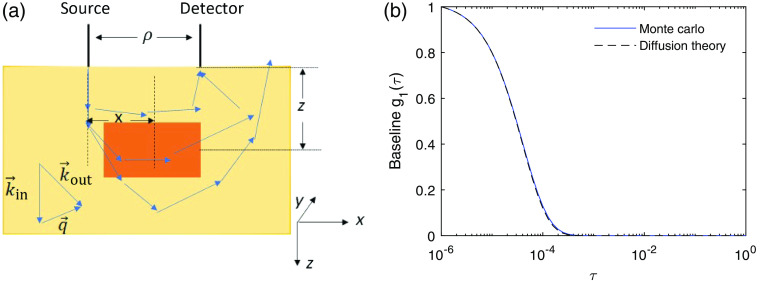
(a) Illustration of the Monte Carlo simulations. For a photon that reaches the detector, the pathlength $L_{n}$ and the accumulated dimensionless momentum transfer $Y_{n}$ were recorded. A local region (shaded with orange color) is specified at z=15  mm, x=15  mm with respect to the position of the source, with size in x and y of 10 mm and size in z = of 4 mm. A detected photon’s total momentum transfer $Y_{n2}$ and pathlength $L_{n2}$ within this local region are also recorded. (b) The representative baseline DCS signal obtained from Monte Carlo simulations as compared with the prediction of diffusion theory obtained from Eq. (4). Here the source–detector separation ρ=30  mm, detector radius r=2  mm, $\mu_{s}^{\prime }=1\;{\mathrm{mm}}^{-1}$ $\mu_{a}=0.01\;{\mathrm{mm}}^{-1}$, $\alpha D=10^{-6}\;{\mathrm{mm}}^{2}/s$, and λ=800  nm.

We used Monte Carlo simulations to model photon migration through a semi-infinite 3D dynamic scattering medium. The Monte Carlo code is a derivation of that utilized in previous publications.^20^^–^^24^ In the simulation, a total number of $10^{8}$ photons were launched on the sample surface and normal to it in the z direction, as illustrated in Fig. 2(a).

The remitted photons were collected at a detector placed ρ=30  mm away from the source and with a radius of r=2  mm. The reduced scattering coefficient of the medium was set to $\mu_{s}^{\prime }=1\;{\mathrm{mm}}^{-1}$. A non-zero $\mu_{a}=0.01\;{\mathrm{mm}}^{-1}$ value was incorporated when calculating the temporal field autocorrelation function $g_{1}(\tau )=\langle E^{*}(t)E(t+\tau )\rangle /\langle {\left|E(t)\right|}^{2}\rangle$ as shown below, where τ is the correlation time and E(t) is the measured electric field at the detector. For the n’th photon arriving at the detector, we recorded its total pathlength $L_{n}$ and the accumulated dimensionless momentum transfer $Y_{n}$. The momentum transfer is the change of the wavevector during a single scattering event $\overset{\to }{q}={\overset{\to }{k}}_{\text{out}}-{\overset{\to }{k}}_{\text{in}}$, where ${\vec{k}}_{\mathrm{in}}$ and ${\vec{k}}_{\mathrm{out}}$ are the incident and scattered wavevectors, respectively. Here, we considered only elastic scattering, thus $|{\overset{\to }{k}}_{\text{in}}|=|{\overset{\to }{k}}_{\text{out}}|=k_{0}$. The accumulated dimensionless momentum transfer of a detected photon is the normalized sum of the square of the momentum transfer of all of the scattering events $Y_{n}=\sum_{s}q_{s}^{2}/2k_{0}^{2}$, where s denotes the s’th scattering event of the n’th photon. With $Y_{n}$ and $L_{n}$ obtained from the Monte Carlo simulations, the temporal field autocorrelation function js calculated as

$$g_{1}(\tau )=\frac{C}{N_{p}}\overset{N_{p}}{\underset{n=1}{\sum }}\;\exp(-\frac{1}{3}\alpha Y_{n}k_{0}^{2}\langle \Delta r^{2}(\tau )\rangle )\exp(-\mu_{a}L_{n}),$$ (1)

where C is a normalization factor such that $g_{1}(0)=1$, $N_{p}$ is the total number of the detected photons, and α is the probability that a scattering event happens at a particular type of scatterer of interest, such as RBCs or neurons. The motion of the scatterers was assumed to be uncorrelated. The functional form of the mean square displacement $\left\langle \Delta r^{2}\right\rangle$ depends on the nature of the dynamics of the scattering particles. For scatterers exhibiting ballistic motion, $\langle \Delta r^{2}(\tau )\rangle =v^{2}\tau^{2}$, where v is the speed of the particles, whereas for diffusive motion $\langle \Delta r^{2}(\tau )\rangle =6D\tau$, where D is the diffusion coefficient. The motion of the RBCs is a combination of ballistic flow and shear-induced diffusion.^25^ It has been experimentally observed and numerically demonstrated that DCS signals are dominated by the diffusive behavior of RBCs.^21^^,^^25^^–^^27^ Thus, we only modeled the contribution from the diffusive behavior of the moving RBCs in this paper. As described later in Secs. 2.2 and 2.3, the dynamics of the neuronal cell motion and vessel wall dilation are modeled as ballistic motions, for we have not seen existing literature that suggests random diffusive behavior of these movements. The baseline DCS signal in the absence of neuronal activation is expressed as

$$g_{1}(\tau )=\frac{C}{N_{p}}\overset{N_{p}}{\underset{n=1}{\sum }}\;\exp(-2\alpha Y_{n}k_{0}^{2}D\tau )\exp(-\mu_{a}L_{n}).$$ (2)

Here, we used $\alpha D=10^{-6}\;{\mathrm{mm}}^{2}/s$, which provides a decay time of $g_{2}$ close to experimental observations,^3^ wavelength λ=800  nm, and $k_{0}=2\pi /\lambda$. The representative baseline DCS signal before neuronal activation that was applied is shown in Fig. 2(b). Experimentally, the intensity temporal autocorrelation function $g_{2}(\tau )=\langle I(t)I(t+\tau )\rangle /{\left\langle |I(t)|\right\rangle }^{2}$ is measured instead of $g_{1}$, and $g_{2}(\tau )$ is related to the theoretically modeled $g_{1}(\tau )$ via the Siegert relation:^28^

$$g_{2}(\tau )=1+\beta g_{1}{\left(\tau \right)}^{2},$$ (3)

with β=1 indicating complete coherence of the detected photons and β<1 accounting for loss of coherence and detection of multiple modes of the electromagnetic field.

The representative DCS signal computed with Monte Carlo simulations is compared with analytical results obtained from the correlation diffusion equation for a semi-infinite medium:^1^^,^^3^

$$g_{1}(\rho ,\tau )=\frac{3C\mu_{s}^{\prime }}{4\pi }[\frac{\exp(Kr_{1})}{r_{1}}-\frac{\exp(Kr_{2})}{r_{2}}].$$ (4)

Here, $K^{2}=3\mu_{a}\mu_{s}^{\prime }+6\mu_{s}^{\prime 2}k_{0}^{2}\alpha D\tau$, $r_{1}={\left(\rho^{2}+z_{0}^{2}\right)}^{1/2}$, $r_{2}={\left(\rho^{2}+{\left(z_{0}+2z_{b}\right)}^{2}\right)}^{1/2}$, $z_{0}=1/\mu_{s}^{\prime }$, and $z_{b}=(5/3)\mu_{s}^{\prime }$. The DCS signals obtained from the Monte Carlo simulation utilizing Eq. (2) and from the theoretical prediction obtained using the correlation diffusion equation [Eq. (4)] are in good agreement as shown in Fig. 2(b).

Neuronal activation can occur in different regions of the brain. Here, we refer to the case of neuronal activation being applied to the full simulation region as global activation. To account for localized neuronal activity within a small region in our model, we specified a second tissue type in the Monte Carlo simulation for a typical local neuronal activation measurement,^29^ with the size in x−y of 10 mm and in z of 4 mm and being 15 mm away from the source in the x direction and 15 mm beneath the sample surface as shown in Fig. 2(a) (orange color), which is utilized to calculate the local neuronal activation that we refer to in this paper. The accumulated dimensionless momentum transfer $Y_{n2}$ and the total pathlength $L_{n2}$ within this local region were also recorded to calculate the DCS signal change induced by neuronal activation only within this local region. The autocorrelation function is then expressed as the contributions from these two tissue types

$$g_{1}(\tau )=\frac{C}{N_{p}}\sum_{n=1}^{N_{p}}\prod_{i=1}^{2}\exp(-2\alpha Y_{ni}k_{0}^{2}D\tau )\exp(-\mu_{a}L_{ni}),$$ (5)

with i=1 and i=2 representing the scattering events and photon trajectories outside and within this local region, respectively, and $L_{n}=L_{n1}+L_{n2}$, $Y_{n}=Y_{n1}+Y_{n2}$.

To quantify the DCS signal variation, we need to identify a parameter that characterizes the decay rate of the $g_{1}(\tau )$ curves. Note that, since the medium is semi-infinite, $g_{1}(\tau )$ is no longer a single exponential decay function, as is the case for an infinite medium. The full expression of Eq. (4) can be used to obtain a blood flow index D. However, a simpler function used for fitting is highly preferred for real-time measurements. The exact solution in Eq. (4) for Brownian motion is recast into $g_{1}(\tau )=\exp(-\tau /\tau_{c})$ when $\tau <\tau_{s}$,^30^ where $\tau_{s}$ is defined as $\tau_{s}=\tau_{c}\sqrt{\left(3/4)\mu_{a}\mu_{s}^{\prime }(\rho^{2}+(z_{0}+z_{b}{)}^{2}\right)}$. For the parameters that we are using, $\tau_{s}/\tau_{c}=2.6$, and $\tau_{c}=46\;\mu s$ is estimated from fitting. We used the first 70  μs of the $g_{1}(\tau )$ curve to fit the functional form of $g_{1}(\tau )=\exp(-\tau /\tau_{c})$, which ensures that $\tau <\tau_{s}$, to obtain the decay time $\tau_{c}$. Finally, we use the single parameter $\tau_{c}$ to characterize the dynamics of the brain tissue.

### 2.2 DCS Signal Change Induced by Neuronal Cell Dynamics

Some studies have demonstrated that action potential propagation induces phase changes of light passing through or reflected from neuronal tissue, corresponding to a membrane displacement on the order of a few nanometers,^12^^,^^31^^–^^35^ which normally happens within 100 ms with respect to the onset of neuronal activation as illustrated in Fig. 1. This phase change is potentially caused by the change of the cell size or the refractive index within the cell. For the purpose of modeling this effect on the DCS signal, we only need to know the effective phase change per unit time, which we discuss in terms of movement of the cell membrane only. We use the average speed of the cell membrane movement of v=1  nm/ms, which is consistent with the literature of *ex vivo* studies of various types of cells^12^^,^^31^^–^^35^ and our recent *in vivo* measurements of the fast optical signals in the mouse brain using optical coherence tomography.^17^ To account for the effect of this neuronal cell motion on the DCS signal, we revised the total temporal field autocorrelation function as

$$g_{1}(\tau )=\frac{C}{N_{p}}\overset{N_{p}}{\underset{n=1}{\sum }}g_{1n,\text{blood}}(\tau )g_{1n,\text{neuronal}}(\tau )\exp(-\mu_{a}L_{n}),\;g_{1n,\text{blood}}(\tau )=\exp(-\frac{1}{3}\alpha Y_{n,\text{blood}}k_{0}^{2}*6D\tau ),\;g_{1n,\text{neuronal}}(\tau )=\exp(-\frac{1}{3}\alpha_{\text{neuronal}}Y_{n,\text{neuronal}}k_{0}^{2}v^{2}\tau^{2}).$$ (6)

Here, C is the normalization factor such that $g_{1}(0)=1$, and $g_{1n}$ denotes the contribution from a single photon. We considered the neuronal scattering probability $\alpha_{\mathrm{neuronal}}$ to be 1, which provides the best-case scenario prediction and $g_{1n,\text{neuronal}}(\tau )=\exp(-\frac{1}{3}Y_{n,\text{neuronal}}k_{0}^{2}v^{2}\tau^{2})$. In reality, this probability can vary with brain regions where the densities of the neurons differ.^36^^–^^38^ However, as discussed in Sec. 3, the induced DCS signal change for this best-case scenario is beyond the sensitivity of currently available DCS systems. Therefore, we do not delve into the details of the variations of the neuronal cell densities in different brain regions in this paper.

### 2.3 DCS Signal Change Induced by Vessel Wall Dynamics

In addition to neuronal cell movement, blood vessels also undergo dynamics due to neurovascular coupling on a slow timescale, as illustrated in Fig. 1. The increase of vessel diameter increases blood volume and decreases vascular resistance, thus resulting in an increase in blood flow. Each of these effects impacts the DCS signal in the following three ways. First, with the blood volume increase, the probability of scattering from the moving RBCs increases and thus α in Eq. (2) increases. The baseline RBC scattering probability is set to be α=2%, which is similar to the volume fraction of the vessels, and we let the scattering probability change in direct proportion to the change in CBV during brain activation. Second, the blood flow speed increases, which causes a proportional increase in the diffusion coefficient D in Eq. (2).^21^ Third, the vessel wall movement results in a phase change of light scattered from the vessel wall. The average speed of the vessel wall movement $v_{\text{vessel}}$ can be obtained from the time course of the vessel diameter change as demonstrated below.

We incorporated the phase changes induced by vessel wall movement (the third effect) into the formalism the same way as the phase changes induced by neuronal cell motion by replacing $g_{1n,\text{neuronal}}$ with $g_{1n,\text{vessel}}(\tau )=\exp(-\frac{1}{3}\alpha Y_{n}k_{0}^{2}v_{\text{vessel}}^{2}\tau^{2})$ in Eq. (6). We found that the magnitude of this (third) effect is at least 5 orders of magnitude smaller compared with that of the blood volume (first) and the flow speed changes (second), and the value of $\tau_{c}$ remains the same for the precision used; thus it is ignored for the rest of the work presented here. We only consider the variation in $g_{1n,\text{blood}}(\tau )=\exp(-\frac{1}{3}\alpha Y_{n,\text{blood}}k_{0}^{2}*6D\tau )$, and $g_{1}(\tau )=\frac{C}{N_{p}}\overset{N_{p}}{\underset{n=1}{\sum }}g_{1n,\text{blood}}(\tau )\exp(-\mu_{a}L_{n})$. To obtain the changes of CBV and CBF, we utilized a single compartment vascular model that considers the full vascular network to be one compartment to estimate the time courses of rCBV(t) and rCBF(t) due to neuronal activation. The letter r denotes the relative value normalized to the baseline value. For example, rCBF(t)=CBF(t)/CBF(0). Here, t=0 denotes the onset of vessel wall dilation. Note that there is a time delay between the onset of vessel wall dilation and the onset of neuronal activation, which is estimated to be about 450 ms,^39^ whereas the time delay for neuronal cell dynamics is typically within 100 ms,^17^ as illustrated in Fig. 1. Thus, when the hemodynamics-induced DCS signal change is considered, the definition of the fast timescale is within 100 ms with respect to the onset of vessel wall dilation or within 450 to 550 ms with respect to the onset of neuronal activation. The functional form of the relative change of the vessel diameter d is given as an input to the model^39^ as follows:

$$rd(t)=d(t)/d(0)=(1+t^{2}\Delta d\;\exp(-t^{2}/\sigma_{d}^{2})).$$ (7)

**Fig. 3:**
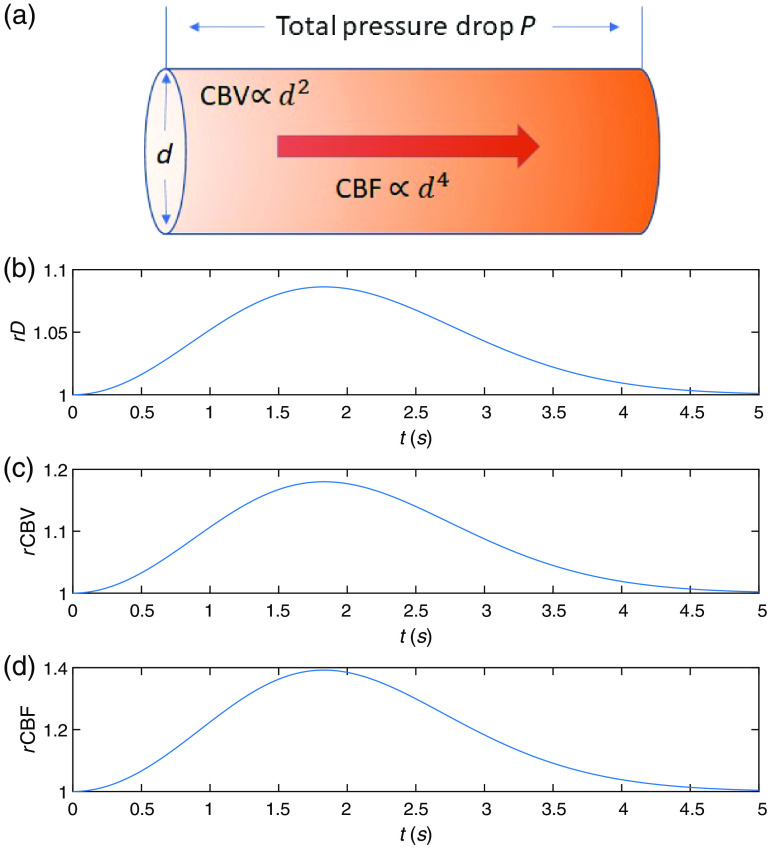
(a) Illustration of the single compartment model of vessel wall dilation process induced by neuronal activation. The brain vasculature is represented as a single vessel with diameter d. The total pressure drop across this vessel is fixed. The relative blood volume rCBV and flow rCBF are related to the relative vessel diameter rd via $r\mathrm{CBF}\propto rd^{4}$ and $r\mathrm{CBV}\propto rd^{2}$. The time courses of (b) relative vessel diameter rd, (c) rCBV, and (d) rCBF after neuronal activation obtained from the single compartment model.

The constant parameters are set to be Δd=0.07 and $\sigma_{d}=1.83\;s$, following the recommended values in Refs. 39 and 40. The time course of the rCBV(t) is related to rd(t) via $r\mathrm{CBV}(t)=rd{\left(t\right)}^{2}$, due to the cylindrical geometry of the vessels and given that the total length of the vessel is invariant. When the total pressure across the compartment is fixed, $r\mathrm{CBF}(t)=rd{\left(t\right)}^{4}$ from Poiseuille’s law. The illustration of the model and the time courses of rd, rCBV, and rCBF are shown in Fig. 3. This is a simpler approximation of the blood flow dynamics following neuronal activation as compared with the multi-compartment model^40^ more commonly used to model fMRI^41^^,^^42^ and fNIRS^18^^,^^43^ measurements. Depending on the hemodynamics model used, the peak value and time of the rCBF(t) and rCBV(t) can vary due to the extra parameter of the delay time between the peak of CBV and CBF in the multi-compartment models. However, for the early time behavior that does not depend on the delay time we are mostly interested in, this simplified model is sufficient, and the magnitude of the results remain the same as compared with using a more sophisticated model.

We explored the DCS signal change due to the variation of α and D within the measurement time T when we start the measurement at time $t_{d}$ after the onset of vessel wall dilation. Note that CBF is the volumetric flow that includes both the effects of the (1) volume and (2) flow speed changes. For the effect of the variation of α and D within T, we consider that, when we start the measurement at a time delay $t_{d}$, the dynamics can be characterized by a time varying diffusion coefficient $D(t_{d},\tau )$ and scattering probability $\alpha (t_{d},\tau )$ with $\alpha (t_{d},\tau )D(t_{d},\tau )=\alpha (t_{d})D(t_{d})(1+R_{r\mathrm{CBF}}\tau )$, where the rate of change is defined as $R_{r\mathrm{CBF}}=\Delta r\mathrm{CBF}/T$, T is the measurement time window, and ΔrCBF is the change of rCBF within T. Strictly speaking, the exact formalism for $\left\langle \Delta r^{2}(\tau )\right\rangle$ in Eq. (1) needs to be calculated from integration for a D value that varies with time. Here, we used a linear approximation as above since the change is small, which can be well described by the first-order term in the Taylor expansion. The product αD at the time delay $t_{d}$ is $\alpha (t_{d})D(t_{d})=\alpha (0)D(0)*r\mathrm{CBF}(t_{d})$. The contribution from a single photon is then $g_{1n,\text{blood}}(\tau )=\exp(-\frac{1}{3}\alpha (t_{d},\tau )Y_{n,\text{blood}}k_{0}^{2}*6D(t_{d},\tau )\tau )$.

We see that the effect of the variation of D and α within the measurement time T to the DCS signal change is a few orders of magnitude smaller compared with the effect of the delay time $t_{d}$ for the range of $t_{d}$ values that we explored, i.e., $t_{d}$ from 50 ms to 2 s. This range of $t_{d}$ is chosen since it provides a DCS signal variation that can be potentially detected using multi-channel DCS systems currently under development. Thus, we have assumed that $\alpha (t_{d},\tau )D(t_{d},\tau )=\alpha (t_{d})D(t_{d})$ for a non-zero $t_{d}$ value used in this paper, leading to $g_{1n,\text{blood}}(\tau ,t_{d})=\exp(-\frac{1}{3}\alpha (0)Y_{n,\text{blood}}k_{0}^{2}*6D(0)*r\mathrm{CBF}(t_{d})\tau )$. Local vessel wall dynamics induced by local neuronal activation were also considered, where the increasing values of α and D and the speed of vessel wall dilation were only applied to the i=2 component in Eq. (5).

### 2.4 Noise Model for DCS Measurements

To determine whether a DCS signal change can be detected experimentally, we compared the DCS signal change induced by neuronal cell movement or hemodynamics with the noise level of the measurement. The noise in $g_{2}(\tau )$ for DCS has been analytically calculated.^30^ The standard deviation of $\left(g_{2}(\tau )-1)\right)$, σ(τ), at each correlation time τ is

$$\sigma (\tau )=\sqrt{T_{b}/T}[\beta^{2}\frac{(1+e^{-2\Gamma T_{b}})(1+e^{-2\Gamma \tau })+2m(1-e^{-2\Gamma T_{b}})e^{-2\Gamma \tau }}{1-e^{-2\Gamma T_{b}}}\;+2{\left\langle n\right\rangle }^{-1}\beta (1+e^{-2\Gamma \tau })+{\left\langle n\right\rangle }^{-2}(1+\beta e^{-\Gamma \tau }){]}^{1/2}.$$ (8)

**Fig. 4:**
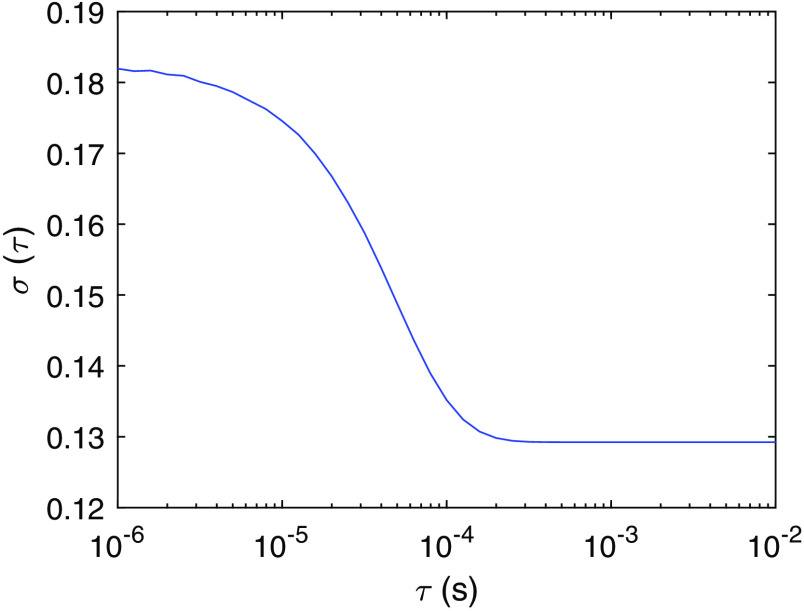
The standard deviation of $\left(g_{2}(\tau )-1\right)$, σ(τ) as a function of correlation time τ obtained from Eq. (8) with the parameters $T_{b}=1\;\mu s$, T=10  ms, β=1, ⟨n⟩=0.1, and $\tau_{c}=46\;\mu s$.

Here, $T_{b}$ is the bin time, m is the bin index, T is the measurement time window, and ⟨n⟩ is the average number of photons within bin time $T_{b}$. The analytical expression $g_{2}(\tau )-1=\beta e^{-2\Gamma \tau }$, where $\Gamma =1/\tau_{c}$. To estimate the noise level in the experiments, we consider the parameters in Eq. (8) to be $T_{b}=1\;\mu s$, T=10  ms, β=1, ⟨n⟩=0.1, and $\tau_{c}=46\;\mu s$, which are obtained from fitting the baseline $g_{1}$ data shown in Fig. 2 using the expression $e^{-\tau /\tau_{c}}$. The resulting σ(τ) is shown in Fig. 4. One way to reduce the noise level is to use multiple channels or multiple instances for averaging. When $N_{c}$ channels are used, the noise level is reduced to $\sigma_{N}(\tau )=\sigma (\tau )/\sqrt{N_{c}}$ from the central limit theorem.

**Fig. 5:**
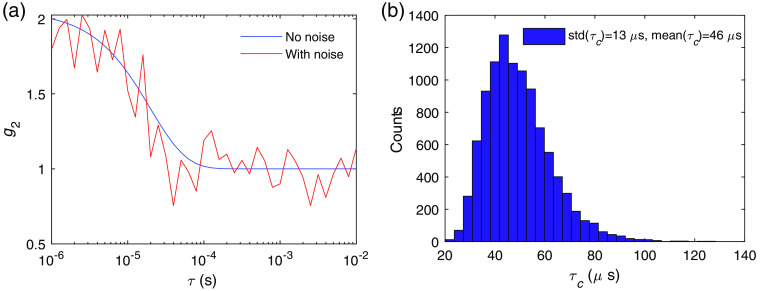
(a) Example of baseline $g_{2}$ with and without adding noise using the DCS system with the noise level as shown in Fig. 4. (b) Distribution of $\tau_{c}$ obtained from fitting using the analytical form $\exp(-\tau /\tau_{c})$ with $10^{4}$ instances of noisy $g_{2}$. The mean and standard deviation of $\tau_{c}$ are indicated in the legend.

**Fig. 6:**
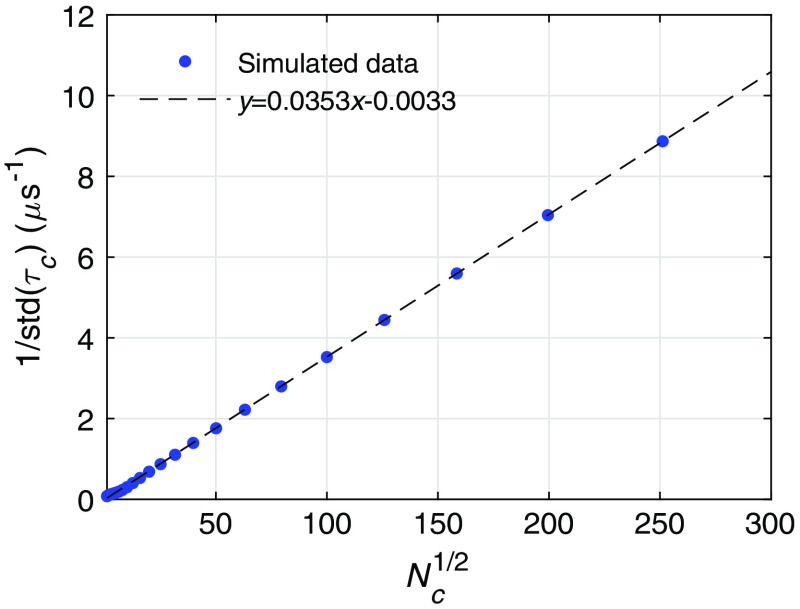
The relation between the noise induced variation of the decay time $\mathrm{std}(\tau_{c})$ numerically computed as a function of the square root of the number of channels/instances used for averaging $N_{c}^{1/2}$. The values of the parameters for the noise model are the same as in Fig. 4, which are $T_{b}=1\;\mu s$, T=10  ms, β=1, ⟨n⟩=0.1, and $\tau_{c}=46\;\mu s$.

Comparison between the noise level and the neuronal activity-induced DCS signal variation determines the CNR of a measurement. However, as discussed, instead of the change of $g_{2}(\tau )$, which is a function of the correlation time τ, a single variable $\tau_{c}$ and its variation are used to characterize the dynamics of the tissue. Thus, the relation between the theoretically calculated σ(τ) and the noise-induced variation of $\tau_{c}$, $\mathrm{std}(\tau_{c})$, is desired. To calculate $\mathrm{std}(\tau_{c})$, we numerically generated noisy $g_{2}(\tau )$ curves by adding random fluctuations drawn from a Gaussian distribution with mean zero and standard deviation σ(τ) at each τ value. An example of the $g_{2}(\tau )$ curves before and after adding noise is shown in Fig. 5(a), using the parameters that give σ(τ) as in Fig. 4. The distribution of the values of $\tau_{c}$ is obtained from the fitting of the noisy $g_{2}(\tau )$ curves, which provides the estimation of $\mathrm{std}(\tau_{c})/\tau_{c}$ induced by noise, as shown in Fig. 5(b). For this particular example $\mathrm{std}(\tau_{c})/\tau_{c}=13\;\mu s/46\;\mu s=0.28$. Thus, this system can only resolve activities that induce a signal change of $\Delta \tau_{c}/\tau_{c}>0.28$. Increasing $N_{c}$ reduces the noise level and thus decreases $\mathrm{std}(\tau_{c})$. We define the CNR of the measurement as $\mathrm{CNR}=\Delta \tau_{c}/\mathrm{std}(\tau_{c})=C*\mathrm{SNR}$, where the relative contrast $C=\Delta \tau_{c}/\tau_{c}$ and the signal-to-noise ratio, $\mathrm{SNR}=\tau_{c}/\mathrm{std}(\tau_{c})$. To estimate the $N_{c}$ required to achieve a CNR ∼1 for neuronal cell movement, vessel wall dilation, and hemodynamics as discussed in Sec. 3, we need to obtain the relation between $\mathrm{std}(\tau_{c})$ and $N_{c}$. Using $\sigma_{N}(\tau )$ to generate noisy $g_{2}(\tau )$ curves, we calculate $\mathrm{std}(\tau_{c})$ as a function of $N_{c}$ as shown in Fig. 6. We see that $1/\mathrm{std}(\tau_{c})$ is proportional to $\sqrt{N_{c}}$. Thus, $\mathrm{CNR}=\Delta \tau_{c}/\mathrm{std}(\tau_{c})$ also increases linearly with $\sqrt{N_{c}}$. This relation determines the required $N_{c}$ to detect an activation, with CNR=1.

## 3 Results

**Fig. 7:**
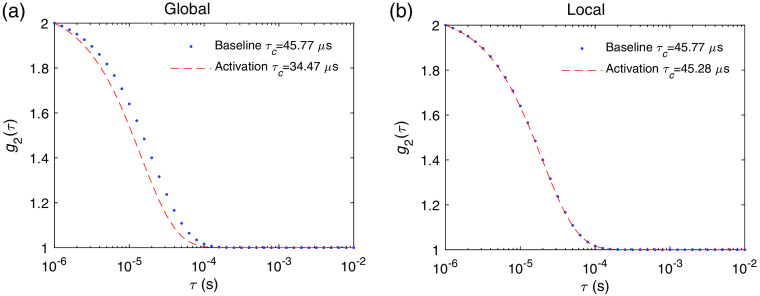
The DCS signal at baseline state $g_{2\text{baseline}}$ and activated state $g_{2\text{activation}}$ induced by hemodynamics for a (a) global neuronal activation, where the variation of $\tau_{c}$ is $\Delta \tau_{c}/\tau_{c}=0.25$, and (b) local neuronal activation, where $\Delta \tau_{c}/\tau_{c}=0.01$. Here, $g_{2\text{activation}}$ is obtained at time t=1.8  s after activation, which corresponds to the peak of the rCBV(t) and rCBF(t) as shown Fig. 3.

In this section, we demonstrate the results of the DCS signal variations induced by neuronal cell motion and hemodynamic changes arising from neuronal activations. We first show the DCS signal change that arises on the slow timescale of a few seconds due to the hemodynamic changes, which is what has been typically measured. As an example, we obtained $g_{2}(\tau )$ at baseline and at the peak of the rCBF(t) response shown in Fig. 3. The results of the induced DCS signal change arising from global and local changes in blood flow are shown in Fig. 7. The decay times $\tau_{c}$ obtained from fitting are indicated in the legends. The induced fractional change of $\tau_{c}$, i.e., $\Delta \tau_{c}/\tau_{c}$, is 0.25 for global and 0.01 for local flow and volume changes. For a given measurement time window T=10  ms, the number of instances/channels required for averaging is $N_{c}=2$ and $N_{c}=3805$ for global and local activation, respectively, to achieve a CNR=1 for the particular set of measurement parameters as used in the noise model in Fig. 4. This can feasibly be detected in real time with the state-of-the-art SPAD cameras.^19^ We can also increase the measurement time T to improve CNR in measurements of the hemodynamics-induced DCS signal changes.

**Fig. 8:**
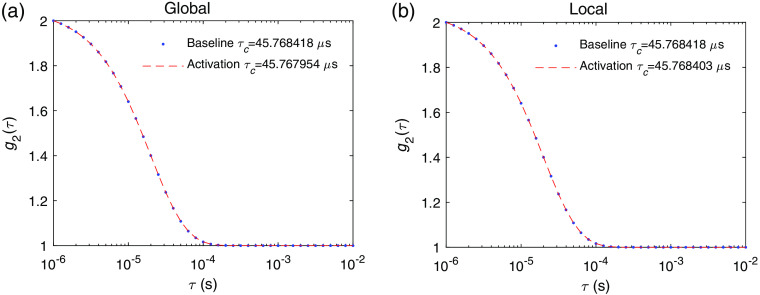
The DCS signal at baseline state $g_{2\text{baseline}}$ and activated state induced by neuronal cell $g_{2\text{activation}}$ for (a) global and (b) local neuronal activation. The variations of $\tau_{c}$ are $\Delta \tau_{c}/\tau_{c}=1.01*10^{-5}$ and $\Delta \tau_{c}/\tau_{c}=3.3*10^{-7}$ for the global and local activation, respectively. The speed of the cell membrane movement used here is 1  nm/ms.

On the fast timescale within 100 ms with respect to neuronal activation, the changes in the DCS signal arise from neuronal cell motion. The results using the speed of the cell membrane movement of 1  nm/ms are shown in Fig. 8. The induced fractional change $\Delta \tau_{c}/\tau_{c}$ is on the order of $10^{-5}$ for global and $10^{-7}$ for local activation. Note that we have assumed that the best-case scenario is all of the scattering events globally or locally happening at a neuronal cell, i.e., we assume α=1. In reality, α≤1, so in reality the induced DCS signal change could be smaller than what we predicted here. Unlike hemodynamics-induced changes that last for a few seconds, the signal induced by neuronal cell motion only lasts for <100  ms.^17^ Thus, increasing the measurement time is not feasible for the detection of neuronal cell motion in real-time measurements. The number of instances/channels required for averaging to achieve CNR ∼1 is $∼10^{9}$ and $∼10^{13}$ for global and local neuronal activation, respectively, for the particular set of measurement parameters as used in the noise model in Fig. 4. This does not seem to be achievable in the near future based on current detector technology trends.^19^

**Fig. 9:**
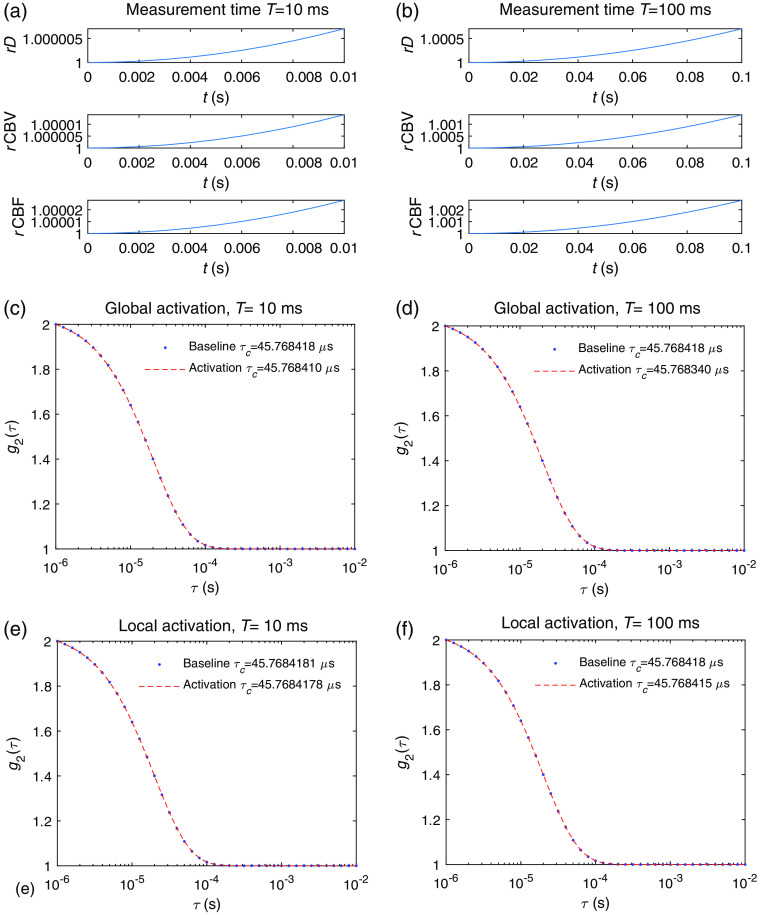
Hemodynamics-induced DCS signal variation for measurements starting at t=0 that corresponds to the onset of vessel wall dilation. The time courses of rD, rCBV, and rCBF for a measurement time (a) T=10  ms and (b) T=100  ms. The baseline $g_{1\text{baseline}}$ and activated $g_{1\text{activation}}$ for a measurement time of (c) T=10  ms, where $\Delta \tau_{c}/\tau_{c}=1.75*10^{-7}$, and (d) T=100  ms, where $\Delta \tau_{c}/\tau_{c}=1.75*10^{-6}$ induced by a global neuronal activation. The baseline $g_{1\text{baseline}}$ and activated $g_{1\text{activation}}$, for a measurement time of (e) T=10  ms, where $\Delta \tau_{c}/\tau_{c}=0.66*10^{-8}$, and (f) T=100  ms, where $\Delta \tau_{c}/\tau_{c}=0.66*10^{-7}$ induced by a local neuronal activation. Note that, in principle, the maximum value of τ will not exceed the measurement time T. We kept the same τ range as the previous figures for easier comparison.

The DCS signal changes induced by fast scale hemodynamics for $t_{d}=0$ (450 ms with respect to the onset of neuronal activation, see Sec. 2.3) were also obtained. Compared with the results of slow signals shown in Fig. 7 in which the measurement took place at the peak of the rCBF(t) curve, we now discuss the results if the measurement took place right after the onset of vessel dilation, i.e., with a measurement time window spanning between t=0 and t=T. Since the functional form of the rCBF increase with time is not linear, the average rate of change $R_{r\mathrm{CBF}}$ varies with measurement time T, as can be seen in Figs. 9(a) and 9(b) for a global activation. The induced change of $\Delta \tau_{c}/\tau_{c}$ is on the order of $10^{-7}$ and $10^{-6}$ for T=10  ms and T=100  ms, respectively, as shown in Figs. 9(c) and 9(d). The results of a local activation are shown in Figs. 9(e) and 9(f), where $\Delta \tau_{c}/\tau_{c}$ is on the order of $10^{-8}$ and $10^{-7}$ for T=10  ms and T=100  ms, respectively. Thus, the induced DCS signal change from fast hemodynamics for $t_{d}=0$ is roughly 1 to 2 orders of magnitude smaller than the effect of neuronal cell on fast timescales, and it cannot be detected by any foreseeable DCS system.

**Fig. 10:**
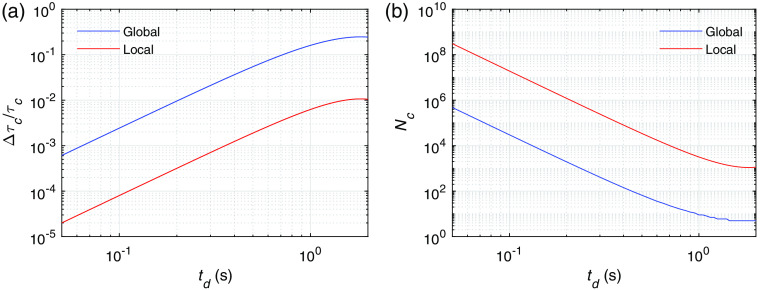
(a) The fractional change of the decay time $\Delta \tau_{c}/\tau_{c}$ as a function of the time delay $t_{d}$ with respect to the onset of vessel wall dilation for global and local neuronal activations. (b) The number of channels $N_{c}$ required to reach a CNR∼1 for the noise level specified as in Fig. 4. The parameters that we utilized are $T_{b}=1\;\mu s$, T=10  ms, β=1, ⟨n⟩=0.1, and $\tau_{c}=46\;\mu s$.

We also calculated the DCS signal variation on slow timescales induced by hemodynamics to estimate the earliest time delay $t_{d}$ with respect to vessel wall dilation that a neuronal activation is measurable for a particular DCS system. As opposed to Fig. 7 where the DCS signal variation is obtained at the peak of the hemodynamic changes, we obtain $\Delta \tau_{c}/\tau_{c}$ measured at different time delays $t_{d}$ with respect to the onset of the vessel wall dilation, i.e., with a measurement time window spanning between $t=t_{d}$ and $t=t_{d}+T$, as shown in Fig. 10(a). For example, at a time delay of 100 ms, $\Delta \tau_{c}/\tau_{c}$ reaches $10^{-2}$ to $10^{-3}$ for global activation and $10^{-3}$ to $10^{-4}$ for local activation. We ignored the DCS signal change induced by changes of D and α within the measurement T, which is at least 4 orders of magnitude smaller. We also estimated the number of channels $N_{c}$ required to reach CNR ∼1 as shown in Fig. 10(b) for the noise level in Fig. 4.

For the current technology that utilizes a kilopixel SPAD camera, for example, it is possible to reach an $\mathrm{SNR}=\tau_{c}/\mathrm{std}(\tau_{c})$ of the order of 300.^19^ To detect the hemodynamic signal, we need $\Delta \tau_{c}/\tau_{c}>\mathrm{std}(\tau_{c})/\tau_{c}=1/\mathrm{SNR}$. From Fig. 10(a), the earliest $t_{d}$ that an activation can be detected is at $t_{d}=100\;\mathrm{ms}$ for global activation or $t_{d}=700\;\mathrm{ms}$ for local activation. Adding the 450 ms, which is the delay time of the onset of vessel wall dilation with respect to neuronal activation (Fig. 1), the earliest time that a neuronal activation is detectable is 550 ms (global) and 1.15 s (local) with respect to the onset of the activation. An even earlier detection of hemodynamic signal can be potentially reached by adopting a megapixel SPAD camera for DCS detection in the future.^44^

## 4 Discussion

We have extensively analyzed the mechanisms that contribute to DCS signals during neuronal activation, including neuronal cell movement and vessel wall movement, though traditionally the variation of the DCS signal is often attributed only to the change of the blood flow, with the diffusion coefficient calculated using Eq. (4). In addition to neurons, other cells such as pericytes and glial cells also reshape due to neuronal activity. Pericytes are contractile cells that act to control the size of the capillaries.^45^^,^^46^ This effect was already incorporated in the hemodynamics as described in Sec. 2.3 since the capillary size change is essentially covered by our modeling of the vascular diameter changes. The glial cells also swell during neuronal activation. However, as the literature suggests, the timescale of the glial cell motion due to the opening of aquaporins is on the order of a few minutes.^9^^,^^47^^–^^49^ This is much slower compared with the effects of neuronal cell motion in response to membrane potential or vessel wall dynamics. We therefore did not model the contributions from pericytes and glial cells dynamics to the DCS signal.

We also obtained the DCS signal variation on both fast and slow timescales. On the fast timescale of 10 to 100 ms after neuronal activation, the largest DCS signal change $\Delta \tau_{c}/\tau_{c}$ induced by neuronal cell motion is on the order of or smaller than $10^{-5}$. This is beyond the sensitivity of any foreseeable DCS system based on current detector technologies due to the large number of channels required to achieve a super high SNR.

**Fig. 11:**
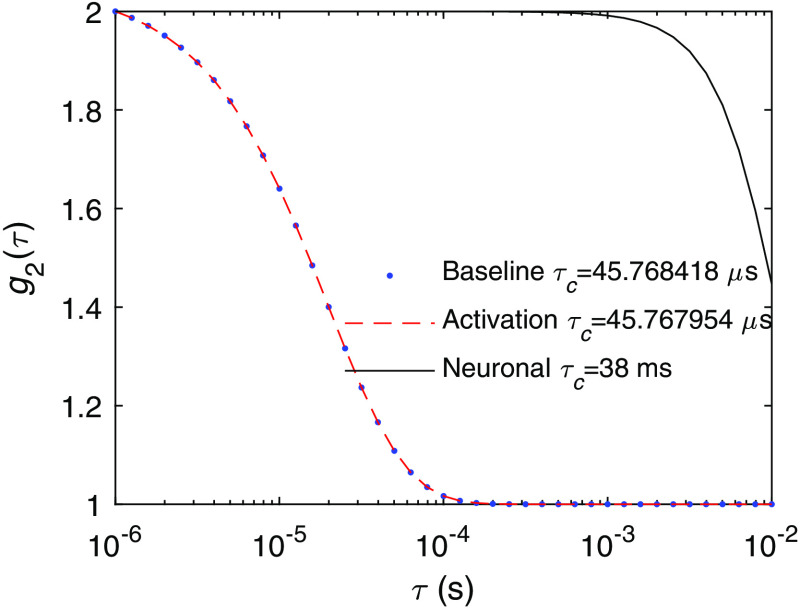
The DCS signal at the baseline and activated states induced by neuronal cell motion as in Fig. 8, compared with the DCS signal induced only by neuronal cell motion without blood flow.

A point worth mentioning is that $\Delta \tau_{c}/\tau_{c}$ is smaller than we had expected. As we can see in Fig. 11, the decay time induced solely by neuronal cell motion is 38 ms, which gives a decay rate $10^{-3}$ that of the decay rate of the baseline DCS signal, but the overall induced $\Delta \tau_{c}/\tau_{c}$ is $10^{-5}$. This is due to the difference in the functional forms of $\left\langle \Delta r^{2}(\tau )\right\rangle$ in Eq. (1), which is $\langle \Delta r^{2}(\tau )\rangle =v^{2}\tau^{2}$ for the ballistic motion of neuronal cell motion and $\langle \Delta r^{2}(\tau )\rangle =6D\tau$ for the diffusive behavior of blood flow. We tested if imposing a diffusive behavior of the neuronal cell motion with the same decay time as in Fig. 11 would induce $\Delta \tau_{c}/\tau_{c}∼10^{-3}$ as expected. This indicates that, if there exists diffusive-like motion associated with neuronal cell motion similar to that of blood flow, the DCS signal change will be 2 to 3 orders of magnitude larger than what was predicted in Fig. 8. To the best of our knowledge, diffusive-like motion has not been reported for neuronal cell motion during activation. The reported time courses of the phase change of light passing through the cells due to activation does not seem to suggest a diffusive behavior.^12^

The change of the DCS signal arising from hemodynamic responses due to neurovascular coupling is another mechanism that can be utilized to detect neuronal activation at a reasonably short latency. We need to add another ∼450  ms to $t_{d}$ in Fig. 10, if the time delay with respect to the onset of the neuronal activation is of interest. This is a slower process compared with cellular motion, but it provides an opportunity to detect sub-second neuronal activation in real time with multi-channel DCS systems.

We analyzed DCS signal changes induced by global and local neuronal activation. The global activation that we refer to here does not have to be an activation that occurs for the whole brain. We define global activation for an activation region large enough to cover the span of most photon trajectories for the particular source–detector separation. For the local activation, we specified a particular small activation region and detection geometry. Apparently, the signal change is different for various geometries, which we did not explore here. We expect the order of magnitude to be similar to what is reported in this work. However, for further exploration, it is straightforward to adjust the parameters in the Monte Carlo simulations to mimic a particular experimental condition. The CNR analysis also depends on the parameters used in the noise model, which can be adjusted accordingly.

We utilized the decay time $\tau_{c}$ as a single parameter to characterize brain dynamics in real time. One limitation for this technique is that it requires the single-photon detector to have high time resolution that is capable of resolving the decay time. In addition to $\tau_{c}$, other parameters such as β, $\mu_{s}$, and $\mu_{a}$^40^ in Eq. (4) and the spatial contrast as measured in laser speckle contrast imaging^50^ may also be utilized to detect neuronal activation. We will compare the performance of using DCS to detect brain activities with other techniques in the future.

In summary, we have extensively analyzed the components of the DCS signal variation during neuronal activation using Monte Carlo simulations. This study has enhanced our fundamental understanding of the underlying mechanisms of brain tissue dynamics that contribute to the DCS signal variations. Our results also provide guidance for the instrument development of DCS systems to detect brain activation with a given latency, which may be relevant for applications such as understanding brain functions, therapeutic monitoring of blood flow, and non-invasive brain–computer interfaces.

## Code, Data, and Materials Availability

The full version of the open-source Monte Carlo code can be found at http://openfnirs.org/software/homer/. The Monte Carlo portion itself is at https://github.com/BUNPC/tMCimg, and another open-source Monte Carlo code that utilizes GPU to accelerate the calculation is at http://mcx.space/. The data that support the results reported in the paper can be requested by contacting the corresponding author.
